# Comparison of Suprascapular Nerve Block With Glenohumeral Joint Dilatation for the Treatment of Adhesive Capsulitis of the Shoulder Joint

**DOI:** 10.7759/cureus.87646

**Published:** 2025-07-10

**Authors:** Madhumita Singha Roy, Anil K Gupta, Dileep Kumar, Sudhir Mishra, Ganesh Yadav, Anit Parihar, Anurug Biswas

**Affiliations:** 1 Department of Physical Medicine and Rehabilitation, King George's Medical University, Lucknow, IND; 2 Department of Radiodiagnosis, King George's Medical University, Lucknow, IND; 3 Department of Physical Medicine and Rehabilitation, Santiniketan Medical College and Hospital, Bolpur, IND

**Keywords:** idiopathic adhesive capsulitis, nerve block, pain, range of motion, shoulder

## Abstract

Background: Adhesive capsulitis (AC) of the shoulder is characterized by progressive pain and loss of active and passive range of motion (ROM). The purpose of this study was to identify a better approach to shoulder pain management and to eliminate unnecessary interventions. The objective was to assess the effect of ultrasound-guided suprascapular nerve block (SSNB), hydrodilatation (HD), and exercise in improving pain, ROM, and function of the shoulder joint.

Method: The study design included a single-blinded randomized controlled trial with an allocation ratio of 1:1:1. The study population consisted of patients attending the OPD and admitted to the ward. A total of 73 patients were analyzed in Group A (Nerve block + Exercise), Group B (Nerve block + HD + Exercise), and Group C (Exercise), using descriptive statistics to make comparisons among the various groups.

Results: The changes were significant (p < 0.001) in all groups, with the maximum change in Group A and the minimum change in Group C. There were fewer significant changes in different ranges of motion, except for flexion, which showed significant improvement at every follow-up.

Conclusion: SSNB plus exercise program not only reduces pain but also reduces disability and improves function, making it an effective treatment for treatment for patients with AC.

## Introduction

Idiopathic adhesive capsulitis (AC) of the shoulder is a disorder of progressive pain and loss of active and passive range of motion (ROM) of the glenohumeral joint. The prevalence rate is 2%-5% [1], affecting people between 40 and 60 years of age with a female predominance [2]. It usually presents spontaneously without any known triggering event, along with the controversy about the most effective treatment approaches. There are many available treatments, but choosing the best out of the most becomes the problem.

Some cases improve with watchful waiting, while others respond to interventions such as injections or exercise. Pain killers and muscle relaxants used orally, exercise, acupuncture, intra-articular steroid injections, nerve blocks, and hydrodilatation (HD) are other available treatment options. Exercise has been shown to improve symptoms with or without the combination of injections. Among others, suprascapular nerve block (SSNB), when used in combination with exercise and saline HD [3], by installation of a large volume of sterile fluid/saline into the glenohumeral joint, is an effective and safe option for restoring shoulder mobility.

The self-limiting nature of this disease affects the clinical decision of patients and care providers who commonly choose to neglect treating AC in favor of "watchful waiting" for the spontaneous resolution, allowing this disease to hamper the functional outcome of patients for a span of two to three years. This study aimed to gain insight into the effectiveness of SSNB, HD, and exercise in combination and as a single modality, thereby assessing the need for multimodal therapies in the management of AC. The primary objective was to achieve pain relief, while the secondary objective was to assess the outcome. To date, no randomized controlled trial has been done taking into consideration the combined effects of three different modalities of treatment as per available literature. The hypothesis was that the combined approach of triple modality would be superior to any of the individual modalities alone in improving the outcomes.

## Materials and methods

All procedures were performed in accordance with the ethical standards of the institutional research committee, following approval obtained from the ethics committee (Ref. code: 97th ECM II B-Thesis/P63) before recruitment, and informed written consent was obtained from all individuals. This was registered in the Clinical Trial Registry of India (Ref. No.: CTRI/2020/06/025885). This prospective, randomized, single-blinded, single-centered, clinical study compared the therapeutic efficacy of ultrasound (US)-guided SSNB by using a solution composed of local anesthetic and triamcinolone with saline HD of the glenohumeral joint using a combination of saline and local anesthetic and no steroid with physical exercise in combination that is SSNB + HD + Physical exercise with SSNB + exercise with Exercise only in the treatment of idiopathic AC.

The inclusion criteria were individuals between the age of 40 and 70 years (male and female) with complaints of spontaneous onset of shoulder pain and stiffness for four weeks along with night pain that was interfering with sleep and painful restriction of both active and passive shoulder abduction and external rotation (ER) of >50% of the normal range with no joint crepitus on examination of the joint and had a normal glenohumeral joint X-ray.

The exclusions were all the patients with a history of trauma to the affected shoulder that resulted in any dislocations, fractures, tear of rotator cuff, clinical evidence of significant cervical spine disorder, osteoarthritis, inflammatory joint diseases and cerebrovascular accidents affecting the shoulder, prior history of corticosteroid injection to shoulder joint within the last three weeks, any surgical interventions, coronary/post-coronary artery bypass surgery, systemic diseases, or medications that would have worsened physical function and interfered with the evaluation of shoulder. This study did not include the patients who had secondary AC like patients with diabetes mellitus (DM), as DM involves an inflammatory response that occurs via several inflammatory mediators with nonenzymatic oxidative reactions between glucose and collagen that results in changes in the microstructural organization of collagen fibers in capsule, which is quite different from the pathophysiology of idiopathic AC, and thus, all the modes of treatment studied here would have exerted different effects in diabetics and nondiabetics.

**Figure 1:**
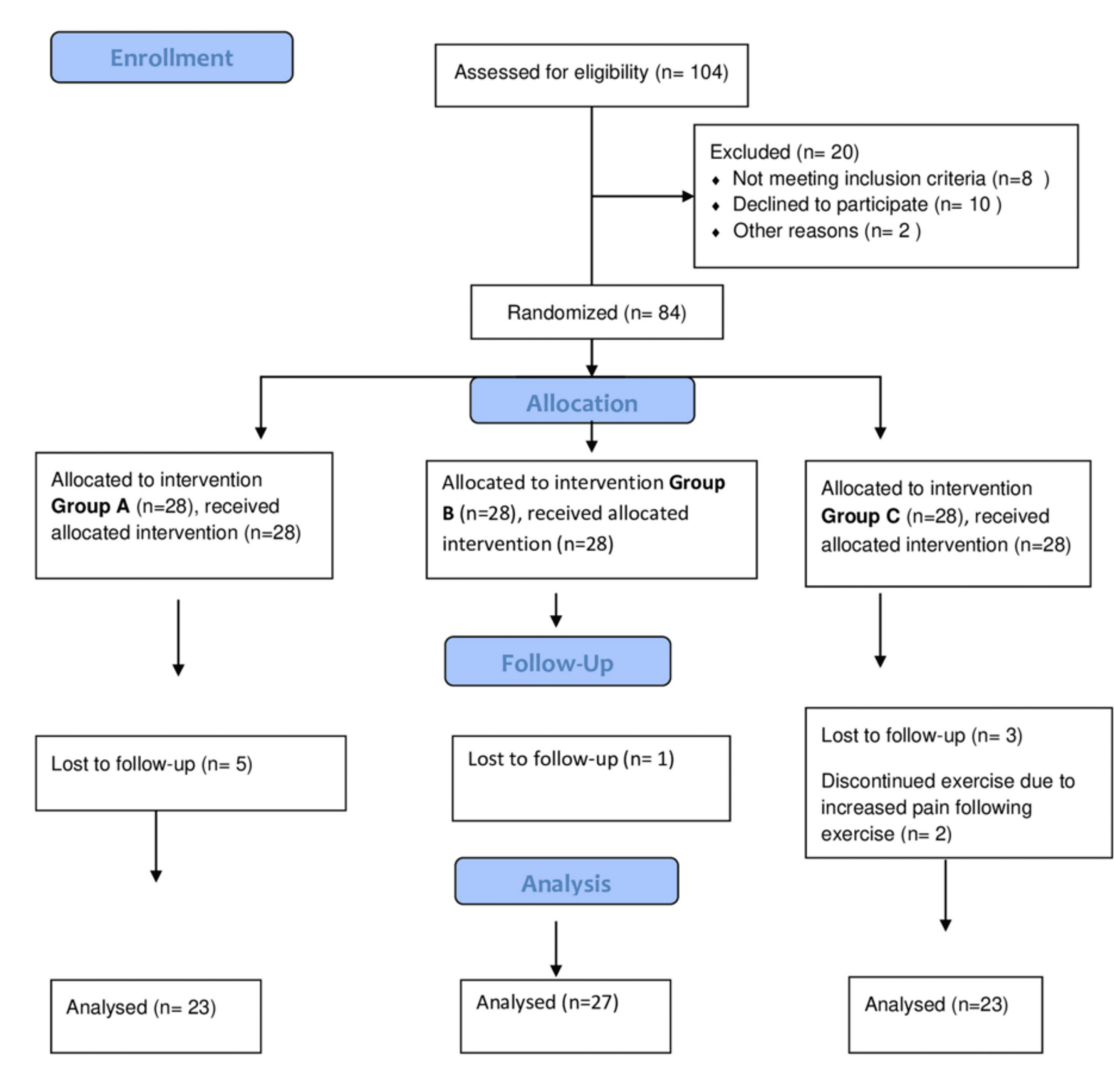
CONSORT flow diagram CONSORT: Consolidated Standards of Reporting Trials

The study was conducted in a tertiary care institution in India. Patients were enrolled according to Consolidated Standards of Reporting Trials randomized controlled trial guidelines (Figure 1).

All enrolled patients underwent assessment according to the working proforma. This included a detailed history, clinical examination, and local examination of the joint with specific tests: 1) Neer’s impingement test, 2) speed test, 3) Yergason test, 4) liftoff test, and 5) apprehension test and radiological examination. Digital X-ray of the involved shoulder joint (anterior-posterior and lateral views) was done to support the diagnosis. Assessment parameters used were the Shoulder Pain and Disability Index (SPADI), the Numeric Pain Rating Scale (NPRS), and goniometry.

NPRS is obtained by asking a patient to rate their pain on an 11-point scale, where 0 and 10 indicate no pain and the worst imaginable pain, respectively. Based on a review of the literature on pain prepared for the Initiative on Methods, Measurement, and Pain Assessment in Clinical Trials II meeting and discussions among the participants, NPRS is recommended as a core outcome measure in clinical trials of pain treatments [4]. In this study, we used this as it is less abstract and easier to understand for the patients.

The patients were randomly divided into three groups using an online random sequence generator by the corresponding author. Enrolment, followed by assigning the patients to interventions, was done by the fourth author (S.M.). The follow-up was conducted by the corresponding author, who was unaware of the groups and remained blinded at the time of data collection during the follow-ups. The outcome assessor was blinded to the study groups. Sample size was calculated based on week 0 and the sixth week variation in SPADI as per a reference study [5]: type I error α = 5% corresponding to a 95% confidence level and type II error β = 20% for detecting results with 80% power of the study were taken. The required sample size (n) was 22 in each group. After adding a 20% loss to follow-up, patients in each group were 26. Eighty-four patients were enrolled in this study. Nine cases lost follow-up, and two cases from Group C discontinued the exercises due to pain and opted for a nerve block.

Treatment procedure

All injections were performed by the primary investigator after a routine US examination of the shoulder. In Group A, SSNB was performed [6] using the US machine, which had a 5-12 Hz linear transducer probe for guidance. The probe was placed in the coronal plane parallel to the spine of the scapula, over the suprascapular fossa, with a slight anterior tilt. The nerve was seen as a round hyperechoic structure at approximately 4 cm depth beneath the transverse scapular ligament in the suprascapular notch, and the suprascapular artery was visualized as a pulsating dot with a positive Doppler signal beside the nerve. Then, a 21 gauge needle was inserted by an in-plane approach after infiltration of skin with 2 mL of 2% lignocaine solution, in a mediolateral direction at an angle of 30°-45° to the vertical, until the needle tip pierced the deep fascia of the supraspinatus muscle/transverse scapular ligament, 6 mL of drug (5 mL of 0.5% bupivacaine + 1 mL/40 mg triamcinolone) was injected slowly into the area around the nerve.

In Group B, after SSNB, HD was performed with a posterior approach [7]. The transducer was then placed parallel and caudal to the scapular spine, after visualizing the joint space between the humeral head and the posterior bony glenoid. Skin infiltration was done as in Group A. Needle of 21 gauge was introduced from lateral to medial direction until the tip reached the junction between the humeral head cartilage and lateral edge of labrum, and 20-30 mL solution depending on patients’ tolerance (15-25 mL 0.9% normal saline and 5 mL 0.5% of bupivacaine) loaded in a 50 mL syringe was injected slowly while gradually advancing the needle to prevent the tip from being ejected backwards due to increased intra articular pressure.

Group C was the control group; patients were advised to follow a home-based exercise program only [8]. The exercise regimen was the same for each group and kept common for each of the participants, advised after the interventions in Groups A and B, and from beginning in Group C. Exercises were shown to patients on the day of their first visit: 1) pendulum exercises, 2) overhead stretch, 3) cross body stretch, 4) ER, and 5) internal rotation (IR) with adduction. Each exercise was performed at a relatively pain-free range for 15-20 seconds, with 10 repetitions each, for a total duration of 20-25 minutes. Exercises were performed two to three times a day, five days a week, for six weeks, with a two-day gap to minimize post-exercise soreness and aid in tissue healing.

**Figure 2:**
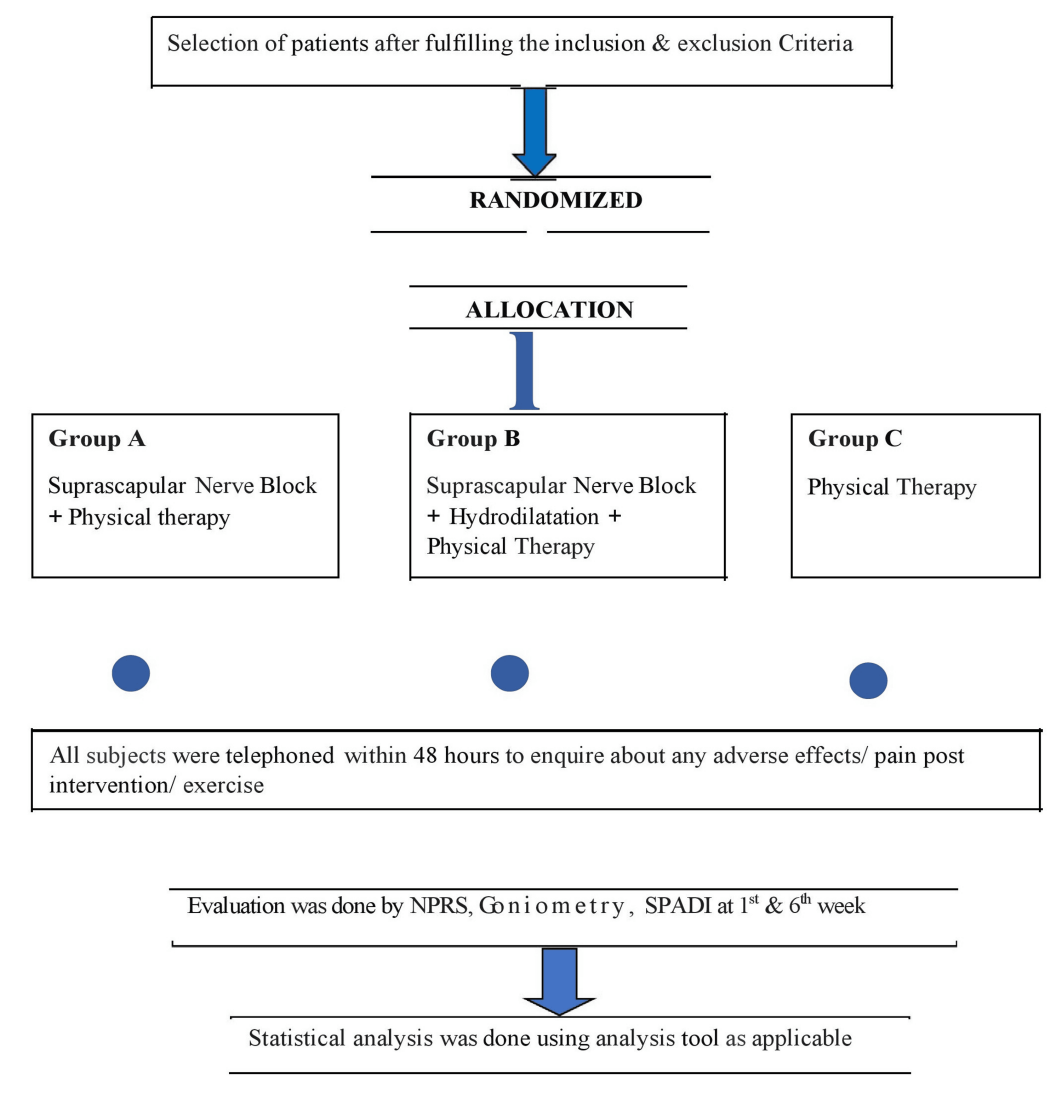
Flowchart of the methodology SPADI: Shoulder Pain and Disability Index; NPRS: Numeric Pain Rating Scale

All patients were advised not to use any other physical modality or medications as part of their treatment during the study period. Subjects were telephoned within 48 hours to ensure that no adverse events had occurred. All three groups consisted of two follow-ups at weeks 1 and 6. The primary outcomes were pain improvement, as measured by the NPRS, and improvement in ROM of the shoulder, as measured by goniometry, at both the first and sixth weeks. The secondary outcome was function and disability assessment by SPADI at the end of six weeks (Figure 2).

The data were analyzed using descriptive statistics and making comparisons among various groups. Categorical data were summarized as proportions and percentages (%), while discrete data were summarized as mean ± standard deviation (SD). An analysis of variance (ANOVA) test was used to compare means among more than two groups. The post hoc Tukey test was applied to compare paired groups.

F-statistic was measured as follows: Mean Squares Treatment / Mean Squares Error or Variation between sample means / Variation within samples. The larger the F-statistic, the greater the variation between sample means relative to the variation within the samples. Thus, the larger the F-statistic, the greater the evidence that there is a difference between the group means. A multivariate linear regression model was employed to evaluate the combined impact of group and comorbidity on study parameters. A two-sided (α = 2) p value <0.05 was considered statistically significant.

## Results

The study examined the distribution of participants across different categories. In terms of the grouping, Group A consisted of 23 individuals, accounting for 31.5% of the total participants, while Group B comprised 27 individuals, representing 37.0%. Group C included 23 individuals, accounting for 31.5% of the participants. Regarding age, the study had a diverse representation. The 40-50 age group included 27 participants, accounting for 37% of the total. The 51-60 age group also consisted of 27 participants, representing 37.0% of the total. The remaining 19 participants fell within the 61-70 years age group, comprising 26% of the sample. Considering gender, there was a relatively balanced representation. The study included 29 male participants, accounting for 39.7% of the total, and 44 female participants, representing 60.3%. In terms of the side involved, 42 participants (57.5%) had the left side affected, while 31 participants (42.5%) had the right side affected. These findings provide insights into the distribution and demographic characteristics of the study participants, contributing to the understanding of the sample composition (Table 1).

**Table 1:** Baseline characteristics of subjects

Variable	Category	n (%)
Group	Group A	23 (31.5)
	Group B	27 (37)
	Group C	23 (31.5)
Age (years)	40-50	27 (37)
	51-60	27 (37)
	61-70	19 (26)
Gender	Male	29 (39.7)
	Female	44 (60.3)
Side involved	Left	42 (57.5)
	Right	31 (42.5)

In the study, participants were divided into three groups: Group A, Group B, and Group C. The ANOVA was conducted to assess the differences among these groups, followed by bicomparisons using p values. The means and SD of the NPRS scores at baseline, first week, and sixth week were recorded for each group. At baseline, the NPRS mean ± SD scores were 6.65 ± 0.88 for Group A, 6.56 ± 1.01 for Group B, and 6.17 ± 1.07 for Group C. The ANOVA revealed no significant difference among the groups (F = 1.51, p = 0.229). Furthermore, the bicomparisons revealed no significant differences between Group A and Group B (p = 0.937), Group A and Group C (p = 0.239), or Group B and Group C (p = 0.371). After one week, the NPRS scores decreased in all groups. The mean ± SD scores were 2.43 ± 1.08 for Group A, 2.33 ± 0.78 for Group B, and 3.7 ± 1.22 for Group C. The ANOVA demonstrated a significant difference among the groups (F = 12.87, p < 0.001). The bicomparisons indicated no significant difference between Group A and Group B (p = 0.936), but significant differences were observed between Group A and Group C (p < 0.001) as well as between Group B and Group C (p < 0.001). At the sixth week, the NPRS scores further decreased. The mean ± SD scores were 2 ± 0.9 for Group A, 1.81 ± 0.88 for Group B, and 2.96 ± 0.98 for Group C. The ANOVA showed a significant difference among the groups (F = 10.68, p < 0.001). The bicomparisons revealed no significant difference between Group A and Group B (p = 0.758), a significant difference between Group A and Group C (p =0.002), and a significant difference between Group B and Group C (p < 0.001). The NPRS scores from baseline to the sixth week showed significant changes for all groups. The ANOVA yielded a significant result for Group A (F = 218.5, p < 0.001), Group B (F = 151.9, p < 0.001), and Group C (F = 57.0, p < 0.001) (Table 2).

**Table 2:** Intergroup and intragroup comparison of NPRS changes NPRS: numeric pain rating scale; ANOVA: analysis of variance; SD: standard deviation

Group	Group A, mean ± SD	Group B, mean ± SD	Group C, mean ± SD	ANOVA	Bicomparisons (p value)		
					Group A vs. Group B	Group A vs. Group C	Group B vs. Group C
NPRS at baseline	6.65 ± 0.88	6.56 ± 1.01	6.17 ± 1.07	F = 1.51, p = 0.229	0.937	0.239	0.371
NPRS in the first week	2.43 ± 1.08	2.33 ± 0.78	3.7 ± 1.22	F = 12.87, p < 0.001	0.936	<0.001	<0.001
NPRS in the sixth week	2 ± 0.9	1.81 ± 0.88	2.96 ± 0.98	F = 10.68, p < 0.001	0.758	0.002	<0.001
NPRS baseline to the sixth week	F = 218.5, p < 0.001	F = 151.9, p < 0.001	F = 57, p < 0.001	-			

In Table 3, the analysis focused on two variables: abduction and flexion. These variables were measured at baseline, first week, and sixth week for Groups A-C. ANOVA was performed to evaluate the differences among the groups, followed by bicomparisons using p values. For abduction at baseline, the mean ± SD values were 100.65 ± 20.13 for Group A, 92.22 ± 8.47 for Group B, and 115.65 ± 19.44 for Group C. The ANOVA demonstrated a significant difference among the groups (F = 12.65, p < 0.001). The bicomparisons revealed significant differences between Group A and Group B (p = 0.177), Group A and Group C (p = 0.008), and Group B and Group C (p < 0.001). In the first week, the abduction values were 119.78 ± 21.45 for Group A, 110.74 ± 13.78 for Group B, and 123.26 ± 20.65 for Group C. The ANOVA result was not statistically significant (F = 3.03, p = 0.055). The bicomparisons showed no significant differences between Group A and Group B (p = 0.210), Group A and Group C (p = 0.803), or Group B and Group C (p = 0.054). During the sixth week, the abduction values were 127.39 ± 23.4 for Group A, 115.93 ± 14.21 for Group B, and 125.87 ± 20.49 for Group C. The ANOVA did not yield a significant result (F = 2.61, p = 0.081). The bicomparisons indicated no significant differences between Group A and Group B (p = 0.102), Group A and Group C (p = 0.962), or Group B and Group C (p = 0.177). The analysis of abduction from baseline to the sixth week showed significant changes in all groups. The ANOVA results were significant for Group A (F = 70.2, p < 0.001), Group B (F = 91.5, p < 0.001), and Group C (F = 35.5, p < 0.001).

**Table 3:** Intergroup and intragroup comparison of shoulder range of motion changes (abduction and flexion) ANOVA: analysis of variance; SD: standard deviation

Variable	Group A, mean ± SD	Group B, mean ± SD	Group C, mean ± SD	ANOVA	Bicomparisons (p value)		
					Group A vs. Group B	Group A vs. Group C	Group B vs. Group C
Abduction at baseline	100.65 ± 20.13	92.22 ± 8.47	115.65 ± 19.44	F = 12.65, p < 0.001	0.177	0.008	<0.001
Abduction in the first week	119.78 ± 21.45	110.74 ± 13.78	123.26 ± 20.65	F = 3.03, p = 0.055	0.210	0.803	0.054
Abduction in the sixth week	127.39 ± 23.4	115.93 ± 14.21	125.87 ± 20.49	F = 2.61, p = 0.081	0.102	0.962	0.177
Abduction baseline to the sixth week	F = 70.2, p < 0.001	F = 91.5, p < 0.001	F = 35.5, p < 0.001	-			
Flexion at baseline	117.17 ± 27.91	96.3 ± 12.14	129.57 ± 23.45	F = 15.07, p < 0.001	0.003	0.137	<0.001
Flexion in the first week	133.04 ± 25.3	110.74 ± 17.02	135.87 ± 23.14	F = 10.12, p < 0.001	0.002	0.900	<0.001
Flexion in the sixth week	135.22 ± 25.91	116.48 ± 18.07	138.26 ± 24.62	F = 6.78, p = 0.002	0.014	0.894	0.004
Flexion baseline to the sixth week	F = 51.1, p < 0.001	F = 54.7, p < 0.001	F = 25.4, p < 0.001	-			

Moving to the variable Flexion, the baseline measurements were 117.17 ± 27.91 for Group A, 96.3 ± 12.14 for Group B, and 129.57 ± 23.45 for Group C. The ANOVA demonstrated a significant difference among the groups (F = 15.07, p < 0.001). The bicomparisons revealed significant differences between Group A and Group B (p = 0.003), Group A and Group C (p = 0.137), and Group B and Group C (p < 0.001). In the first week, the Flexion values were 133.04 ± 25.3 for Group A, 110.74 ± 17.02 for Group B, and 135.87 ± 23.14 for Group C. The ANOVA result was significant (F = 10.12, p < 0.001). The bicomparisons showed significant differences between Group A and Group B (p = 0.002), Group A and Group C (p = 0.900), and Group B and Group C (p < 0.001). During the sixth week, the Flexion values were 135.22 ± 25.91 for Group A, 116.48 ± 18.07 for Group B, and 138.26 ± 24.62 for Group C. The ANOVA result was significant (F = 6.78, p = 0.002). The bicomparisons indicated significant differences between Group A and Group B (p = 0.014), Group A and Group C (p = 0.894), and Group B and Group C (p = 0.004). The analysis of Flexion from baseline to the sixth week showed significant changes in all groups. The ANOVA results were significant for Group A (F = 51.1, p < 0.001), Group B (F = 54.7, p < 0.001), and Group C (F = 25.4, p < 0.001). The analysis focused on two variables, ER and IR, as shown in Table 4.

**Table 4:** Intergroup and intragroup comparison of shoulder range of motion changes (external and internal rotations) ANOVA: analysis of variance; SD: standard deviation

Variable	Group A, mean ± SD	Group B, mean ± SD	Group C, mean ± SD	ANOVA	Bicomparisons (p value)		
					Group A vs. Group B	Group A vs. Group C	Group B vs. Group C
External rotation at baseline	16.09 ± 7.68	13.7 ± 7.28	21.3 ± 7.72	F = 6.47, p = 0.003	0.510	0.056	0.002
External rotation in the first week	22.61 ± 7.21	21.3 ± 7.28	26.09 ± 8.78	F = 2.47, p = 0.092	0.823	0.288	0.083
External rotation at the sixth week	25.43 ± 8.11	23.52 ± 7.44	28.48 ± 9.59	F = 2.19, p = 0.119	0.700	0.438	0.100
External rotation baseline to sixth week	F = 75.2, p < 0.001	F = 100.6, p < 0.001	F = 23.7, p < 0.001	-			
Internal rotation at baseline	33.26 ± 9.25	35.19 ± 5.63	35.65 ± 6.79	F = 0.7, p = 0.498	0.623	0.510	0.972
Internal rotation in the first week	38.7 ± 8.01	40 ± 5.72	39.35 ± 6.27	F = 0.24, p = 0.79	0.771	0.941	0.937
Internal rotation in the sixth week	39.57 ± 8.11	41.11 ± 4.87	41.3 ± 5.68	F = 0.54, p = 0.587	0.664	0.619	0.994
Internal rotation baseline to sixth week	F = 37.7, p < 0.001	F = 38.3, p< 0.001	F = 24.7, p < 0.001	-			

These variables were measured at baseline, the first week, and the sixth week for Groups A-C. ANOVA was performed to evaluate the differences among the groups, followed by bicomparisons using p values. For ER at baseline, the mean ± SD values were 16.09 ± 7.68 for Group A, 13.7 ± 7.28 for Group B, and 21.3 ± 7.72 for Group C. The ANOVA demonstrated a significant difference among the groups (F = 6.47, p = 0.003). The bicomparisons revealed no significant differences between Group A and Group B (p = 0.510), but significant differences were observed between Group A and Group C (p = 0.056) as well as between Group B and Group C (p = 0.002). In the first week, the ER values were 22.61 ± 7.21 for Group A, 21.3 ± 7.28 for Group B, and 26.09 ± 8.78 for Group C. The ANOVA result was not statistically significant (F = 2.47, p = 0.092). The bicomparisons showed no significant differences between Group A and Group B (p = 0.823), Group A and Group C (p = 0.288), or Group B and Group C (p = 0.083). During the sixth week, the ER values were 25.43 ± 8.11 for Group A, 23.52 ± 7.44 for Group B, and 28.48 ± 9.59 for Group C. The ANOVA did not yield a significant result (F = 2.19, p = 0.119). The bicomparisons indicated no significant differences between Group A and Group B (p = 0.700), Group A and Group C (p = 0.438), or Group B and Group C (p = 0.100). The analysis of ER from baseline to the sixth week showed significant changes in all groups. The ANOVA results were significant for Group A (F = 75.2, p < 0.001), Group B (F = 100.6, p < 0.001), and Group C (F = 23.7, p < 0.001). Moving to the variable IR, the baseline measurements were 33.26 ± 9.25 for Group A, 35.19 ± 5.63 for Group B, and 35.65 ± 6.79 for Group C. The ANOVA demonstrated no significant difference among the groups (F = 0.7, p = 0.498). The bicomparisons showed no significant differences between Group A and Group B (p = 0.623), Group A and Group C (p = 0.510), or Group B and Group C (p = 0.972). In the first week, the IR values were 38.7 ± 8.01 for Group A, 40 ± 5.72 for Group B, and 39.35 ± 6.27 for Group C. The ANOVA result was not statistically significant (F = 0.24, p = 0.79). The bicomparisons showed no significant differences between Group A and Group B (p = 0.771), Group A and Group C (p = 0.941), or Group B and Group C (p = 0.937). During the sixth week, the IR values were 39.57 ± 8.11 for Group A, 41.11 ± 4.87 for Group B, and 41.3 ± 5.68 for Group C. The ANOVA did not yield a significant result (F = 0.54, p = 0.587). The bicomparisons indicated no significant differences between Group A and Group B (p = 0.664), Group A and Group C (p = 0.619), or Group B and Group C (p = 0.994). The analysis of IR from baseline to the sixth week showed significant changes in all groups. The ANOVA results were significant for Group A (F = 37.7, p < 0.001), Group B (F = 38.3, p < 0.001), and Group C (F = 24.7, p < 0.001). The analysis focused on two variables, the SPADI Pain Score and the SPADI Disability Score, as shown in Table 5.

**Table 5:** Intergroup and intragroup comparison of SPADI pain and disability scores SPADI: Shoulder Pain and Disability Index; ANOVA: analysis of variance; SD: standard deviation

Variable	Group A, mean ± SD	Group B, mean ± SD	Group C, mean ± SD	ANOVA	Bicomparisons (p value)		
					Group A vs. Group B	Group A vs. Group C	Group B vs. Group C
SPADI pain score at baseline	31.61 ± 5.06	32.78 ± 3.87	29.65 ± 3.92	F = 3.32, p = 0.042	0.604	0.276	0.033
SPADI pain score in the first week	13.74 ± 4.56	12.19 ± 4.12	19.48 ± 5.22	F = 16.6, p < 0.001	0.467	<0.001	<0.001
SPADI pain score at the sixth week	9.3 ± 4.78	8.78 ± 3.49	14.22 ± 5.1	F = 10.79, p < 0.001	0.909	0.001	<0.001
SPADI pain baseline to the sixth week	F = 383.0, p < 0.001	F = 452.6, p < 0.001	F = 151.6, p < 0.001	-			
SPADI disability score at baseline	54.13 ± 8.08	56.52 ± 5.62	52.91 ± 3.91	F = 2.29, p = 0.108	0.355	0.777	0.100
SPADI disability score in the first week	41.52 ± 10.69	44.81 ± 9.38	45.52 ± 5.35	F = 1.37, p = 0.261	0.390	0.279	0.957
SPADI disability score at the sixth week	36.04 ± 12.62	37.63 ± 10.79	41.26 ± 7.09	F = 1.52, p = 0.227	0.854	0.215	0.442
SPADI disability baseline to the sixth week	F = 58.5, p < 0.001	F = 90, p < 0.001	F = 90.1, p < 0.001	-			

These variables were measured at baseline, first week, and sixth week for Groups A-C. ANOVA was performed to evaluate the differences among the groups, followed by bicomparisons using p values. For the SPADI Pain Score at baseline, the mean ± SD values were 31.61 ± 5.06 for Group A, 32.78 ± 3.87 for Group B, and 29.65 ± 3.92 for Group C. The ANOVA demonstrated a significant difference among the groups (F = 3.32, p = 0.042). The bicomparisons revealed no significant differences between Group A and Group B (p = 0.604), but significant differences were observed between Group A and Group C (p = 0.276) as well as between Group B and Group C (p = 0.033). At the first week, the SPADI Pain Score values were 13.74 ± 4.56 for Group A, 12.19 ± 4.12 for Group B, and 19.48 ± 5.22 for Group C. The ANOVA result was statistically significant (F = 16.6, p < 0.001). The bicomparisons showed no significant differences between Group A and Group B (p = 0.467), but significant differences were observed between Group A and Group C (p < 0.001) as well as between Group B and Group C (p < 0.001). During the sixth week, the SPADI Pain Score values were 9.3 ± 4.78 for Group A, 8.78 ± 3.49 for Group B, and 14.22 ± 5.1 for Group C. The ANOVA yielded a significant result (F = 10.79, p < 0.001). The bicomparisons indicated no significant differences between Group A and Group B (p = 0.909), but significant differences were observed between Group A and Group C (p = 0.001) as well as between Group B and Group C (p < 0.001). The analysis of the SPADI Pain Score from baseline to the sixth week showed significant changes in all groups. The ANOVA results were significant for Group A (F = 383.0, p < 0.001), Group B (F = 452.6, p < 0.001), and Group C (F = 151.6, p < 0.001).

Moving to the variable SPADI Disability Score, the baseline measurements were 54.13 ± 8.08 for Group A, 56.52 ± 5.62 for Group B, and 52.91 ± 3.91 for Group C. The ANOVA did not yield a significant difference among the groups (F = 2.29, p = 0.108). The bicomparisons showed no significant differences between Group A and Group B (p = 0.355), Group A and Group C (p = 0.777), or Group B and Group C (p = 0.100). In the first week, the SPADI Disability Score values were 41.52 ± 10.69 for Group A, 44.81 ± 9.38 for Group B, and 45.52 ± 5.35 for Group C. The ANOVA result was not statistically significant (F = 1.37, p = 0.261). The bicomparisons showed no significant differences between Group A and Group B (p = 0.390), Group A and Group C (p = 0.279), or Group B and Group C (p = 0.957). During the sixth week, the SPADI Disability Score values were 36.04 ± 12.62 for Group A, 37.63 ± 10.79 for Group B, and 41.26 ± 7.09 for Group C. The ANOVA did not yield a significant result (F = 1.52, p = 0.227). The bicomparisons indicated no significant differences between Group A and Group B (p = 0.854), Group A and Group C (p = 0.215), or Group B and Group C (p = 0.442). The analysis of the SPADI Disability Score from baseline to the sixth week showed significant changes in all groups. The ANOVA results were significant for Group A (F = 58.5, p < 0.001), Group B (F = 90, p < 0.001), and Group C (F = 90.1, p < 0.001).

## Discussion

Idiopathic AC of the shoulder joint involves synovial inflammation, leading to capsular fibrosis [9], usually starting without any specific precipitating event. This study had the majority of patients aged between 40 and 60 years, and only 26% were between 61 and 70 years. A study by Santia et al. [10] also supported the common age for AC between 40 and 60 years and stated that the incidence declines after the sixth decade of life, in both men and women. It has a female preponderance [11], and pathogenesis in women during their perimenopause period may be driven by hormonal factors, especially by the low estrogen levels, as evidence suggests that estrogen has inflammation-dampening effects [12]. AC was seen more commonly involving the left shoulder, as right-handedness is more predominant [13]. The SSNB solution used in this study contained LA+steroid, which reduced pain. Since Group C did not receive any pain medications, the pain arising from the liberation of pain mediators by the exercise contributed to the maximum NPRS. Chang et al. [14] found that SSNB provided better pain relief compared with exercise alone and suggested that US should be used as a guidance tool, which was similar to the method and the results of our study. Studies have used a similar SSNB solution, with the concept that triamcinolone has systemic anti-inflammatory effects. The duration of the benefits of nerve blockage lasts longer than the pharmacologic effects of the drug due to desensitization and decreased peripheral nociceptive input in the dorsal horn [15].

**Figure 3:**
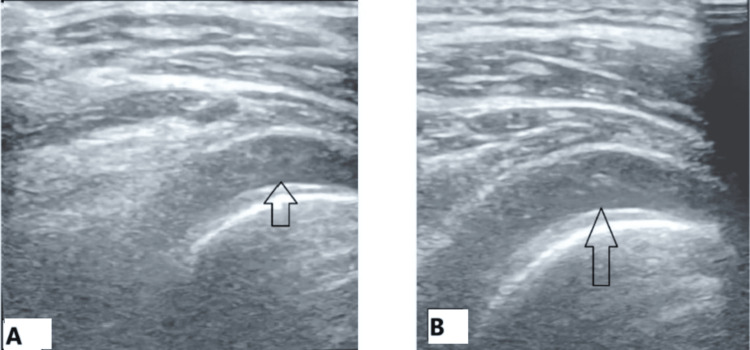
(A) Hydrodilatation of the right shoulder joint. (B) Joint space in ultrasound before dilatation. A significant increase in the joint space after hydrodilatation is shown

Previous studies reported that HD is a relatively effective way to provide direct results and fast recovery, if used in the frozen phase when there is slight or moderate rigidity and greater elasticity of the capsule results in a more effective distending effect of the normal saline [16]. Allen [17] stated that capsule rupture does not necessarily occur through the areas of maximum adhesions, and the pressure cannot increase further to stretch the capsule if rupture occurs. In this study, we used US guidance that helped us monitor the fluid accumulation in real time and prevented any incidence of capsule rupture as confirmed by the absence of fluid extravasation; thus, all patients in Group B had sustained elevated tension in their shoulder joint space and more effective stretching and pain improvement (Figure 3).

Subramanyam et al. [18] reported similar sustained improvements in pain scores with HD. Studies that used steroids in the HD solution had unclear results, as steroids can reduce the pain, so the separate roles of saline and local anesthetics could not be estimated from those studies. This is in contrast to the solution used in this study, as it did not contain a steroid; however, the sustained benefits suggest that HD can be effective with normal saline and local anesthetic alone. Vad et al. [19] used a similar HD solution, and the results were also comparable. This study had only one sitting of HD, as studies suggested that repeated dilation does not have any added effect [20].

In a study conducted by Chan et al. [21], it was found that patients in stage 2/stage 3 of AC respond better to exercise, and exercise programs are considered the first-line therapy. The study by Diercks and Stevens [22] suggested that gentle therapy is better than intensive therapy. As the exercise program advised in our study was gentle and performed within the available pain-free range, Group C patients also had significant and maintained pain improvement starting from the first day of exercise until the end of the study period. However, these improvements were less than those of the other two groups, as Groups A and B had a combined approach that targeted multiple factors of the disease pathology.

The thickened coracohumeral ligament and posterior capsular tightness have been responsible for limiting ER and IR of the shoulder, respectively. Adhesions of the axillary fold to itself and to the anatomical neck of the humerus are responsible for abduction restriction; thus, it can be assumed that these planes of motion showed less improvement than the shoulder flexion, as none of the procedures used in this study directly targeted the above-mentioned pathological processes.

Shoulder motion and pain reduction are important factors in decreasing the physical disability of patients with AC. No difference in SPADI Disability score among the three groups in any of the follow-ups is directly related to the activities of daily living function, which was affected due to the reduced abduction and rotation of the shoulder. Shanahan et al. [23] showed improvement in the SPADI Score until four weeks after SSNB, after which there was a decline, which is in contrast with our study, as improvements were maintained until the end of six weeks. During the bigroup comparisons, there were significant differences in pain, shoulder ROM, and SPADI parameters between Groups A and C and Groups B and C until the last follow-up, but no difference was there between Groups A and B in any of the follow-ups. It shows that the result does not change with the application of three modalities together, as per the presumed hypothesis; simply a nerve block followed by an exercise program can sufficiently bring about the same change in the outcomes. As HD cannot reduce the fibrosis and only targets the weaker areas of the capsule, the force generated by it can only partially improve pain and disability; thus, no patient in this study had complete resolution of symptoms.

However, it was a very short-duration study, as many patients were from rural parts of the state. After the pain relief, follow-up was discontinued; hence, the long-term effects of SSNB and HD could not be assessed from this study. Moreover, blinding was not possible for the patients, nor for the primary investigator, so the chances of bias could not be eliminated entirely. This study did not include diabetics, so the majority of patients were missed in the comparisons made here; thus, the generalization of results was not possible. The use of steroids in the SSNB might be responsible for the prolonged pain relief and thus maximum changes, whereas the absence of steroids in HD could be responsible for the less significant changes.

## Conclusions

Joint HD and SSNB are both effective in combination. The nerve block is performed without entering the joint cavity as an outpatient procedure with fewer adverse effects than HD, as HD has postprocedural pain issues, and there is a greater need for analgesics after HD. Patients were also not able to start the exercises immediately due to discomfort.

Thus, SSNB, with significantly less postprocedural pain and a minimal need for analgesics, can be used preferentially. If followed by an adequate exercise regimen composed of stretching, strengthening, and mobilization exercises, it yields satisfactory outcomes. However, more high-quality studies with a longer follow up period on a much larger as population are required to establish the best modality for the management of idiopathic AC.
